# Descriptive study of stress and satisfaction at work in the Saragossa university services and administration staff

**DOI:** 10.1186/1752-4458-4-7

**Published:** 2010-04-21

**Authors:** Jose Miguel Tricas Moreno, Carlos Salavera Bordas, Ma Orosia Lucha Lopez, Concepcion Vidal Peracho, Ana Carmen Lucha Lopez, Elena Estebanez de Miguel, Luis Bernues Vazquez

**Affiliations:** 1 Physiotherapy Research Unit, Zaragoza University, Zaragoza, Spain; 2 Education Faculty, Zaragoza University, Zaragoza, Spain; 3 Division of Endocrinology and Metabolism, Royo Villanova Hospital, Zaragoza, Spain

## Abstract

**Background:**

The notion of stress in connection with the work environment became an important topic during the 1970's, when the first studies on the subject were published and the term of work stress was first coined. In 1974, Freudenberger proposed the term *burnout *to refer to the condition of physical and emotional exhaustion, as well as the associated negative attitudes, resulting from the intense interaction in working with people. The aim of our study is to examine burnout and job satisfaction in Saragossa University Services and Administration Staff (SAS) and detect the main factors which could contribute to too much stress, because job stress has emerged as a major psychosocial influence on mental health, associated with burnout.

**Methods:**

24 people from the Services and Administration Staff in the University of Saragossa participated in the study. The research was carried out during the implementation of a module on Stress Management organised by the University of Saragossa and commissioned to the Unit for Research in Physical Therapy (University School of Health Sciences) from that University. This research is an exploratory research to improve the stress management program. A personal interview was carried out and additionally, participants were given the Maslach Burnout Inventory and the Scale of Satisfaction at Work of Warr, Cook & Wall.

**Results:**

However using small sample this is worth to state that participants present most of them low burnout levels in the burnout scale. Only in one person high exhaustion level was reflected, even though other seven showed mean levels; in the professional self-esteem section, most of them showed high self-esteem, with two cases of low self-esteem and five with mean level.

With regard to satisfaction people participating in the study show mean levels in intrinsic as much as in extrinsic factors and general satisfaction.

**Conclusions:**

Services and Administration Staff from the University of Saragossa shows low burnout levels linked with high professional self-esteem and low emotional exhaustion and depersonalization.

It has been found also medium levels in work satisfaction probably related with the continuous quality improvement efforts in the academics environment to create protective factors in decreasing levels of job stress.

These results show that not only personality or temperament have an influence on burnout and stress, also the job conditions are related with these diseases. These aspects should be taken into account in the design of stress prevention programme at work.

## Background

Contrary to popular belief, stress is not necessarily harmful. In fact, having stress means being alive, although stress is usually not associated with pleasure, satisfaction or success. The concept of "health and stress" was first proposed by Dr Hans Selye [1], who defined it as the way in which our body responds to the demands of our life. In keeping with this definition, dealing with stress does not mean having to eliminate stress, but rather learning to handle or administer it. Stress is a protective response. In certain occasions it can be beneficial, as it allows us to cope with and overcome difficult situations that require all our energy. In addition, in many situations stress can even make us feel good.

The main question however is: what are the negative effects of stress? Stress results in health deterioration, decrease in performance and a dysfunctional way of dealing with things. The notion of stress in connection with the work environment became an important topic during the 1970's, when the first studies on the subject were published and the term of work stress was first coined. In 1974, after observing a large number of volunteers working with him in an alternative health care institution, Freudenberger proposed the term *burnout *[2] to refer to the condition of physical and emotional exhaustion, as well as the associated negative attitudes, resulting from the intense interaction in working with people [3-6]. Burnout could be considered as stress syndrome related with professional and institutional stress [7] and defined as a combination of three components: the environment, negative thoughts, and physical responses [8]. In high stress situations, a number of symptoms are indicative of the presence of this syndrome: low self-esteem, emission of defective judgement, feeling of being overwhelmed, lack of memory for details, inability to concentrate, loss of perspective, emotional and more irrational thought, irritable and unpredictable behaviour, changes in communication styles, an increase in the number of mistakes while on duty, fatigue and greater negative self-criticism [9]. Furthermore, employees experiencing burnout may easily become angry and irritated, exhibit stubbornness and inflexible thinking, abuse substances, express cynicism about the agency, and increase increasingly less productive [10]. Maslach and Jackson [11], for example, have conceptualized burnout as encompassing the tripartite components of emotional exhaustion, depersonalization, and reduced personal accomplishment. Specifically, emotional exhaustion refers to the feelings of being emotionally drained by intense contact with other people; depersonalization refers to the negative attitude or callous responses toward people; and reduced personal accomplishment refers to a decline in one's sense of competence and of successful achievement in working with people.

A number of stressors have been associated with burnout; organizational stressors stemming from the organizational structure, climate, and management style are an important part of them. Although such stressors in the workplace sometimes cannot be entirely circumvented, when the number and types experienced exceed the individual's ability to cope with them comfortably, job stress can make an employee susceptible to burnout [12,13]. One of the primary buffers against job stress and burnout is perceived organizational social support [14].

Particular concerns have been expressed about the high levels of physical and mental problems, including burnout, among health care workers, teachers and sportsmen [15-20], but little research has been found in administrative and service staff, thought it could be also considered a caring profession with lot of responsibility in daily running of institutions.

By reason of this, the aim of our study is to examine burnout and job satisfaction in Saragossa University Services and Administration Staff (SAS) and detect the main factors which could contribute to too much stress, because job stress has emerged as a major psychosocial influence on mental health, associated with burnout. Like some previous research suggests that individual coping responses do not alleviate strain produced by job stress, institutions should take actions to reduce stress among employees [21] and a study like this could be a way of communication that is a basic feature of any effective and cooperative work relationship.

## Methods

### Procedure

Tests were carried as the first step of a module on Stress Management organised by the University of Saragossa and commissioned to the Unit for Research in Physical Therapy (University School of Health Sciences) from that University. To know the burnout level and the satisfaction at job of the staff allowed the personal of the Unit for Research in Physical Therapy to adapt the design of the Stress Management Module. A personal interview was carried out in order to collect sociodemographic data and to ascertain the reason which had prompted them to seek medical consultation. Additionally, participants were given the above mentioned questionnaires on work satisfaction [22] and the MBI [23]. Participation in the study entailed undertaking later treatment and was voluntary in nature. Montero & León [24] guidelines were followed for the implementation of the ex post facto prospective study.

The protocol was fully explained to each subject before they signed an informed consent document. The submitted study conformed to the policies established by the Code of Ethics of the World Medical Association (Declaration of Helsinki).

Participants were randomized between the workers of the Services and Administrative Staff of the Zaragoza University included in the Stress Management module developed by the Unit for Research in Physical Therapy (University School of Health Sciences) in 2009. Nobody had important somatic or psychological desease. Everybody had employment involving direct attention to teachers and students.

Reliability of the scales was obtained with Cronbach's coefficient (Table 1). Cronbach's alpha will generally increase as the intercorrelations among test items increase. Because intercorrelations among test items are maximized when all items measure the same construct, Cronbach's alpha is widely believed to indirectly indicate the degree to which a set of items measures a single unidimensional latent construct. Thus, whereas the modal intercorrelation among test items will equal zero when the set of items measures several unrelated latent constructs, the average intercorrelation among test items will be greater than zero in this case.

**Table 1:** Reliability of the scales with Cronbach's alpha coefficient

	Cronbach's alpha	Elements
Warr	,855	16
Maslach	,894	16

### Participants

27 people from the Services and Administration Staff in the University of Saragossa participated in the study, coming from 9 representative centres of San Francisco Campus in Zaragoza University. Only 24 cases, those who started and completed the study, were considered valid. The remaining fills in only few items and we have eliminated questionnaires. This is a preliminary prospective study to measure the level of burnout of the services and administration staff in Zaragoza University to adapt stress intervention to reality, so the sample size is fairly small, which may reduce the statistical power. 16.7% (N = 4) of the people participating in the study and later treatment were men and 83.3% (N = 20) were women (See additional file 1). Ages range between 27 and 62 years, with an average age of 45.04 years (SD = 8.595) (See additional file 2). The average weekly time devoted to the project was 35 hours. 16.8% (N = 4) of the participants held positions of responsibility, 58.3% (N = 14) performed secretarial jobs and 24.9% (N = 6) fulfilled jobs requiring physical effort at the University (See additional file 3). With regards to job permanence, 20.8% (N = 5) of them had spent less than 5 years working at University, 12.6% (N = 3) between 5 and 10 years; 33.3% (N = 8) between 10 and 20 years; and the remaining 33% (N = 8) had spent over 20 years in the job (See additional file 4).

### Research materials

- Personal interview: a one-hour interview was carried out prior to the inclusion of each participant in the study. Sociodemographic data was collected (age, sex, educational attainment, marital status, origin, place of employment, years of permanence at University and years of work in general) with the aim of gathering variables which could correlate with stress factors as well as for finding markers that were significant to the objective of our study.

- Maslach Burnout Inventory [23], in its version for professionals in "human services" (MBI-HSS). We have choose the MBI-HSS instead of the MBI-GS, because everybody included in the study have a direct contact with the client or user of the service, and as it is an important burnout source we have decided to priories this information using the MBI-HSS. It consists of 22 items which are answered using a Likert scale. Informants indicated the frequency with which they had experienced feelings and attitudes described in each item using a number from 0 (never) to 6 (every day). The questionnaire assesses aspects such as emotional exhaustion (EE), depersonalization and personal accomplishment (PA). A person obtaining high scores in EE and depersonalization and low ones in PA is considered to suffer from the syndrome.

- Scale of Satisfaction at work of Warr, Cook and Wall [22]. There are increasing number of studies addressing dissatisfaction and turnover to burnout [25,26]. To measure the satisfaction at work of our subjects we have used the Scale of Satisfaction at work of Warr, Cook and Wall. It covers overall job satisfaction and satisfaction with nine aspects of work, each rated on a seven point Likert scale with higher scores representing greater satisfaction, reflecting the experience of workers in a remunerated job [15,22]. It focuses on the informant's affective response to the content of their job. The scale measures the general satisfaction and it is in line with the position of scholars establishing a dichotomy of factors and it is designed to encompass both the intrinsic and the extrinsic aspects of job conditions. It consists of two subscales:

- Subscale for intrinsic factors: it deals with aspects such as the recognition obtained from one's work, responsibility, promotion, aspects related to the task content, etc. This scale consists of four items: "Freedom to choose your own method of working", "Recognition you get for good work", "Amount of responsibility you are given", "Opportunity to use your abilities", "Amount of variety in your job".

- Subscale for extrinsic factors: it investigates workers' satisfaction with aspects related to the organization of work, such as timetables, wages, environmental conditions at work, etc. This scale consists of five items: "Physical working conditions", "Your colleagues and fellow workers", "Your remuneration", "Your hours of work".

### Statistical analyses

For the statistical analysis, cases were reviewed, leaving out those which had not been completed (N = 3).

Descriptive statistics, such as mean, standard deviation and percentage were used to summarize the subject's data and the answer of the inventory and the scale. The correlation of data was tested by using Spearman correlation analysis.

The statistical tests were performed using the statistical software package SPSS (Statistical Package for the Social Sciences), version 15.0 (LEAD Technologies, Athens, GA, USA).

## Results

The analysis of the MBI questionnaire (Maslach Burnout Inventory) reveals in the emotional exhaustion scale (Table 2. Exhaustion level) that 66.7% (N = 16) of the participants in the study showed low exhaustion levels, 29.2% (N = 7) presented medium exhaustion levels and only 4.2% (N = 1) reflected a high level, which may be related to labour stress and burnout scores.

**Table 2:** 

		Frequency	Percentage	Cumulative Percentage
**EXHAUSTION LEVEL**	Low emotional exhaustion level	16	66.7	66.7
	Medium emotional exhaustion level	7	29.2	95.8
	High emotional exhaustion level	1	4.2	100.0
	Total	24	100.0	100.0

**DEPERSONALIZATION**	Low depersonalization	21	87.5	87.5
	Mean depersonalization	2	8.3	95.8
	High depersonalization	1	4.2	100.0
	Total	24	100.0	100.0

**PERSONAL ACCOMPLISHMENT**	Low professional self-esteem	2	8.3	8.3
	Mean professional self-esteem	5	20.8	29.2
	High professional self-esteem	17	70.8	100.0
	Total	24	100.0	100.0

With regard to depersonalization (Table 2. Depersonalization), the results obtained in the questionnaire indicate lower levels of stress than the ones obtained when examining exhaustion level. In fact, 87.5% (N = 21) of the participants show scores which indicate low depersonalization, while 8.3% (N = 2) showed medium levels and only one person, (4.2%) showed high depersonalization scores, with stress labour punctuations, which correspond to burnout levels.

The analysis of the level of personal accomplishment (Table 2. Personal Accomplishment) shows that 70.8% (N = 17) of the participants had high professional self-esteem levels at the moment of the study, while self-esteem was weaker in the remaining 29.2%. 20.8% (N = 5) of these cases reported medium levels and 8.3% (N = 2) showed low levels of professional self-esteem.

The questionnaire on job satisfaction by Warr, Cook and Wall measures general satisfaction, extrinsic satisfaction and intrinsic satisfaction.

With reference to extrinsic satisfaction, the results indicate that 4% (N = 1) of the participants in the study show low satisfaction, 65.6% (N = 16) medium satisfaction and 29.2% (N = 7) high satisfaction with respect to those factors related to the external conditions of their work (colleagues, physical conditions, superiors...).

The data obtained for intrinsic factors reveals that low satisfaction levels were found in 12% (N = 3) of the participants, whereas medium levels were found in 65.6% (N = 16) of the cases and high scores in the remaining 20.9% (N = 5). These results indicate high levels of satisfaction relative to these internal factors (acknowledgement, responsibility, promotion opportunities, etc.).

In relation to general satisfaction only 4.2% (N = 1) showed low values, while values were medium in 70.7% (N = 16) of the cases and high in 25.1% (N = 7) of them, which suggests high general satisfaction as regards work, including both intrinsic and extrinsic factors.

The correlation between the two questionnaires (MBI and Job Satisfaction) by establishing the extent to which the factors included in one of the scales correlated with those in the other scale could help to detect the main factors which could contribute to too much stress.

The Spearman correlation rate was calculated. You can see the values of Spearman's rho in Table 3 (See additional file 5). Correlations with p values lower than 0.01 are marked with ** and p values lower than 0.05 are marked with *. Intrinsic factors were found to correlate significantly with extrinsic factors (0.706), with emotional exhaustion (-0.544) and with depersonalization (-0,497). Among sociodemographic factors, years of actual employment correlates significantly with emotional exhaustion (0,444). Moreover previous employment correlates with intrinsic factors (0,783), extrinsic factors (0,677) and emotional exhaustion (-0,672). Finally, significant correlation was also found between work hours and years of working (0.432), but any aspect related with burnout correlated with work hours, sex or place of employment.

As we can see from the above results, extrinsic factors, intrinsic ones, emotional exhaustion and depersonalization show high correlation degrees. Due to this relationship between variables, it may be hypothesized that by treating one of these factors, the other correlated factors will be affected. However, work hours and place of employment scarcely correlate with the rest, so modifying one of these variables will not have a significant effect on the rest of variables.

## Discussion

The central aim of the present study was to examine work stress (burnout) and job satisfaction among Services and Administration Staff from the University of Saragossa. The results show low burnout levels and medium levels in general satisfaction and also in both intrinsic and extrinsic factors.

The levels of burnout in this study are lower than in other studies where about one fourth of the participants [27] or one third of the participants had symptoms of burnout [28]. Although previous studies have demonstrated that contexts involving regular, significant, and chronically stressful interpersonal contact and also boundary-spanners, like we have studied, are especially susceptible to burnout [29,30], our sample doesn't show high burnout levels. Most of them show high professional self-esteem level, and low levels in emotional exhaustion and low depersonalization which is related with not burnout [31,32]. These results could be explained through autonomy although it wasn't a point of this research. Several studies confirm that this variable is a necessary component of employee empowerment, alleviating lodging employee's emotional exhaustion and the depersonalization [30,33,34].

High levels in the exhaustion and in the depersonalization dimension of burnout are strongly associated with the musculoskeletal, cardiovascular and other physical diseases [27] so the low levels in these dimensions in our study could protect the participants from suffering that kind of diseases. Some studies [35] have shown that social support is effective in reducing burnout, so programmes as the proposed in this study, refining social support into supervisory, coworker, and content-specific supportive measures shows great promise, also for the prevention of associated illness.

The medium levels in general satisfaction and in both intrinsic and extrinsic factors that we have found in our study could be explained by the greatest predictors of job satisfaction in the academic community related to the environment in academics work, including university atmosphere, morale, sense of community and relationships with colleagues [36]. This is also related with the high correlation shown between extrinsic factors and the general satisfaction. As predicted, burnout was highly correlated with variables as job dissatisfaction; desire to quit the job, physical and emotional symptoms and perceived performance level. Implications for differential treatment of stress and burnout are offered suggesting that the focus in treating burnout should be on enhancing people's sense of their work's importance and significance [37].

Though the rates of sex are not equal between men and women, this study doesn't show any gender differences in burnout. Recent studies [38,39] have shown that gender differences in the use of coping strategies may be decreasing and becoming less consistent over the past two decades, and this may parallel social changes in gender roles and constraints. Perhaps these social changes explain the small effect of gender differences in this study.

The present study had many limitations, including the small sample size, which might have reduced the statistical power and the difficulty of generalizing the present findings based on a specific sample of administrative staff in Zaragoza University. The sample does not provide the opportunity to examine influences of visible diversity such as gender and race due to the dominance of female Caucasian respondents. Also, the cross-sectional design does not allow examination of the conceptual model variables over time or causal inferences. To truly isolate the underlying causal linkages of job stress, and burnout, a longitudinal design is needed. Future research that incorporates reciprocal relationships longitudinally could lead to organizational changes that promote better client, employee, and organizational outcomes.

## Conclusions

Services and Administration Staff from the University of Saragossa shows low burnout levels linked with high professional self-esteem and low emotional exhaustion and depersonalization.

It has been found also medium levels in work satisfaction probably related with the continuous quality improvement efforts in the academics environment to create protective factors in decreasing levels of job stress.

The results demonstrate that not only personality or temperament have an influence on burnout and stress, also the job conditions are related with these diseases. These aspects should be taken into account in the design of stress prevention programme at work.

This study had not take into account the possible relationship between burnout and other pathologies, but it is important to highlight that detection and prevention of burnout can avoid the development of other important problems of public health.

## Competing interests

The authors declare that they have no competing interests.

## Authors' contributions

JMTM made the conception and participated in the design of the study. CSB participated in analysis and interpretation of data. MOLL contributed to the acquisition of data and in the draft and revision of the manuscript. CVP participated in the design and coordination of the study and the revision of the manuscript. ACLL contributed to the acquisition of data and in the draft and revision of the manuscript. EEM contributed to the acquisition of data and in the draft and revision of the manuscript. LBV participated in the coordination of the study and the revision of the manuscript. All authors read and approved the manuscript.

## Supplementary Material

Additional file 1 **Figure 1: Distribution of sex**. the file contains a graphic showing the population's distribution of sex. Click here for file

Additional file 2 **Figure 2: Distribution of age**. the file contains a graphic showing the population's distribution of age. Click here for file

Additional file 3 **Figure 3: Place of employment**. the file contains a graphic showing the place of employment of the population. Click here for file

Additional file 4 **Figure 4: Job permanence**. the file contains a graphic showing the job permanence of the population. Click here for file

Additional file 5 **Table 3: Correlations**. The file contains a table with the Spearman correlation rate between the most important variables of the study. Click here for file
