# Supplementary material for: Descriptive study of stress and satisfaction at work in the Saragossa university services and administration staff
Source: Int J Ment Health Syst. 2010 Apr 21;4:7. doi: 10.1186/1752-4458-4-7 (PMC2873570; doi:10.1186/1752-4458-4-7)
Supplement: Additional file 1 — Figure 1: Distribution of sex. the file contains a graphic showing the population's distribution of sex. [file 1752-4458-4-7-S1.DOC]

0

5

10

15

20

25

men

women
