# Supplementary material for: Descriptive study of stress and satisfaction at work in the Saragossa university services and administration staff
Source: Int J Ment Health Syst. 2010 Apr 21;4:7. doi: 10.1186/1752-4458-4-7 (PMC2873570; doi:10.1186/1752-4458-4-7)
Supplement: Additional file 3 — Figure 3: Place of employment. the file contains a graphic showing the place of employment of the population. [file 1752-4458-4-7-S3.DOC]

positions of

responsibility

secretarial jobs

jobs requiring physical

effort
