# Supplementary material for: Descriptive study of stress and satisfaction at work in the Saragossa university services and administration staff
Source: Int J Ment Health Syst. 2010 Apr 21;4:7. doi: 10.1186/1752-4458-4-7 (PMC2873570; doi:10.1186/1752-4458-4-7)
Supplement: Additional file 4 — Figure 4: Job permanence. the file contains a graphic showing the job permanence of the population. [file 1752-4458-4-7-S4.DOC]

<5 years

Between 10-20

years

0

1

2

3

4

5

6

7

8

<5 years

Between 5-10 years

Between 10-20 years

>20 years
