# Supplementary material for: Descriptive study of stress and satisfaction at work in the Saragossa university services and administration staff
Source: Int J Ment Health Syst. 2010 Apr 21;4:7. doi: 10.1186/1752-4458-4-7 (PMC2873570; doi:10.1186/1752-4458-4-7)
Supplement: Additional file 5 — Table 3: Correlations. The file contains a table with the Spearman correlation rate between the most important variables of the study. [file 1752-4458-4-7-S5.RTF]

	Age 	Sex 	Place of employment 	Work hours 	Years of actual employment	Years working	Previous employment	Intrinsic factors	Extrinsic factors	Emocional exhaustion	Depersonalization	
Sex	-,202											
Place of employment	-,451(*)	,021										
Work hours 	,260	,012	,098									
Years of actual employment	,594(**)	-,202	-,316	,393								
Years working	,801(**)	-,118	-,384	,432(*)	,571(**)							
Previous employment	,284	-,169	,016	-,060	-,079	-,053						
Intrinsic factors	-,232	,003	,330	-,193	-,127	-,214	,783(*)					
Extrinsic factors	-,242	-,099	,277	-,106	-,343	-,274	,677(*)	,706(**)				
Emotional exhaustion	,251	-,099	-,298	,367	,444(*)	,247	-,321	-,544(**)	-,613(**)			
Despersonalization 	-,075	,316	-,082	,009	-,188	,001	-,672(*)	-,497(*)	-,144	-,078		
Personal accomplishment	-,169	-,265	-,003	-,256	-,185	-,239	-,198	-,341	-,083	,044	,209	
